# Polyphenol Rich *Ajuga bracteosa* Transgenic Regenerants Display Better Pharmacological Potential

**DOI:** 10.3390/molecules26164874

**Published:** 2021-08-11

**Authors:** Samina Rubnawaz, Waqas Khan Kayani, Nosheen Akhtar, Rashid Mahmood, Asif Khan, Mohammad K. Okla, Saud A. Alamri, Ibrahim A. Alaraidh, Yasmeen A. Alwasel, Bushra Mirza

**Affiliations:** 1 Department of Biochemistry, Faculty of Biological Sciences, Quaid-i-Azam University, Islamabad 45320, Pakistan; bushramirza@qau.edu.pk; 2 Department of Biotechnology, Faculty of Sciences, University of Kotli, Azad Jammu and Kashmir 11100, Pakistan; wkkayani@gmail.com; 3 Department of Biological Sciences, National University of Medical Sciences, Rawalpindi 46000, Pakistan; nosheenakhtar@numspak.edu.pk; 4 Drugs Control & Traditional Medicines Division, National Institute of Health, Islamabad 45320, Pakistan; alrashidoon@gmail.com; 5 Institute of Biological Sciences, Faculty of Sciences, University of Malaya, Kuala Lumper 50603, Malaysia; asif.khan.qau@gmail.com; 6 Botany and Microbiology Department, College of Science, King Saud University, Riyadh 11451, Saudi Arabia; malokla@ksu.edu.sa (M.K.O.); saualamri@ksu.edu.sa (S.A.A.); ialaraidh@ksu.edu.sa (I.A.A.); Yasmeen@ksu.edu.sa (Y.A.A.)

**Keywords:** *Ajuga bracteosa*, genetic transformation, antioxidants, polyphenols, metabolic profiling, RP-HPLC, pharmaceutical properties, BALB/c mice

## Abstract

*Ajuga bracteosa* Wall. ex Benth. is an endangered medicinal herb traditionally used against different ailments. The present study aimed to create new insight into the fundamental mechanisms of genetic transformation and the biological activities of this plant. We transformed the *A. bracteosa* plant with *rol* genes of *Agrobacterium rhizogenes* and raised the regenerants from the hairy roots. These transgenic regenerants were screened for in vitro antioxidant activities, a range of in vivo assays, elemental analysis, polyphenol content, and different phytochemicals found through HPLC. Among 18 polyphenolic standards, kaempferol was most abundant in all transgenic lines. Furthermore, transgenic line 3 (ABRL3) showed maximum phenolics and flavonoids content among all tested plant extracts. ABRL3 also demonstrated the highest total antioxidant capacity (8.16 ± 1 μg AAE/mg), total reducing power, (6.60 ± 1.17 μg AAE/mg), DPPH activity (IC50 = 59.5 ± 0.8 μg/mL), hydroxyl ion scavenging (IC50 = 122.5 ± 0.90 μg/mL), and iron-chelating power (IC50 = 154.8 ± 2 μg/mL). Moreover, transformed plant extracts produced significant analgesic, anti-inflammatory, anticoagulant, and antidepressant activities in BALB/c mice models. In conclusion, transgenic regenerants of *A. bracteosa* pose better antioxidant and pharmacological properties under the effect of *rol* genes as compared to wild-type plants.

## 1. Introduction

Medicinal plants have been used in traditional medicines for thousands of years and they contain a wide variety of biologically active plant products called secondary metabolites. In the last decade, the potential toxicity of synthetic drugs led to a resurgence of the use of these metabolites as a facile and economical alternative approach [1]. According to the World Health Organization (WHO), approximately, 80% population of developing countries depends on plant products to alleviate and treat serious ailments [2].

Free radicals, such as reactive oxygen and nitrogen species, produced in the human body can be a root cause of aging, rheumatism, malignancies, diabetes, cardiovascular, and neurodegenerative disorders [3]. A higher rate of incidence of these diseases triggered the research of natural antioxidants and various studies have suggested that dietary polyphenols are the most abundant antioxidants in nature [4]. These polyphenols can directly scavenge free radicals, chelate metal ions, and inhibit their pro-oxidant activities, thus reducing the risk of chronic metabolic diseases. Moreover, polyphenols also possess anti-inflammatory, anti-thrombotic, and analgesic activities [5]. Likewise, different trace elements not only improve plant immunity but also provide scaffolding for antioxidant enzymes as cofactors in humans. Hence, it is a dire need to thoroughly investigate different herbal extracts for their antioxidant properties to develop new drugs [6].

*Ajuga bracteosa* is considered an elixir to a variety of ailments and it is found in hilly areas of Pakistan, Nepal, Kashmir, India, and the Himalayan region. An extensive literature survey reveals that different extracts of *A. bracteosa* have a variety of pharmacological activities. It is used to cure skin infections, respiratory issues, digestive problems, malaria, protozoal diseases, diabetes mellitus, hepatitis, arthritis, epilepsy, inflammation, neurological disorders, and cancer [7,8,9,10]. This medicinal importance is due to a repertoire of metabolites such as essential oils, ecdysteroids, terpenoids, phenolics, flavonoids, and withanolides characterized in this plant [11].

*Root oncogenic loci* (*rol*) genes are well known for the upregulation of secondary metabolism [12]. Different *rol* genes have different induction capacities of secondary metabolites [13] and a lot of the pharmacological activities are linked to the number of secondary metabolites produced in the medicinal plants [14]. We hypothesize that the amount of these secondary metabolites biosynthesized de novo in *A. bracteosa* could be enhanced and hence the better pharmacological effects could be attained. Considering that, we transformed *A. bracteosa* with *rolABC* genes of *Agrobacterium* *rhizogenes*, and hairy roots were produced. To resolve the issue of organ specificity of certain metabolites, intact plants (regenerants) were regenerated from these transgenic hairy root lines and their extracts were screened for antioxidant activities. Multiple in vivo activities including analgesic, anti-inflammatory, antidepressant, and anticoagulant activity was evaluated using BALB/c mice. In the continuation of our previous study [15], estimation of essential elements of transformed regenerated plants of *A. bracteosa* by atomic absorption spectrophotometry and HPLC fingerprinting of polyphenol content was performed to support our results.

## 2. Results

### 2.1. Elemental Analysis

The elemental screening of transformed and untransformed *A. bracteosa* digested extracts revealed the presence of 10 elements, commonly found in plants, as listed in Table 1. Data shows that transgenic line 3 (ABRL3) is enriched with 3 macro-elements: sodium 3.94 ± 2 µg/mg dry weight (DW), potassium 13.09 ± 2 µg/mg DW, and calcium 1.95 ± 0.5 µg/mg DW. While magnesium is more abundant in transgenic line 1 (2.15 ± 0.3 µg/mg DW). Likewise, the highest amounts of all micro-elements are found in ABRL3 except chromium which is more prevalent in ABRL1. On the other hand, untransformed wild type (WT) plant extract has the least concentrations of all elements ranging from 0.004 ± 0.01 µg/mg to 8.06 ± 0.3 µg/mg of dry sample. We also observed that cadmium and lead are below the detection limit in all samples.

### 2.2. Qualitative Screening

Qualitative analyses showed the presence of medicinally important phytoconstituents in the methanol: chloroform extracts of *A. bracteosa* summarized in Table 2. These findings suggest that alkaloids, phenolics, flavonoids, and glycosides are present in all extracts in a larger amount. Whereas tannins and saponins are found in moderate amounts. Anthocyanin, β-cyanins, coumarins, and sterols were absent in all extracts while terpenoids were identified only in transgenic lines.

### 2.3. Quantitative Analyses

#### 2.3.1. Determination of TPC and TFC

**Figure 1:**
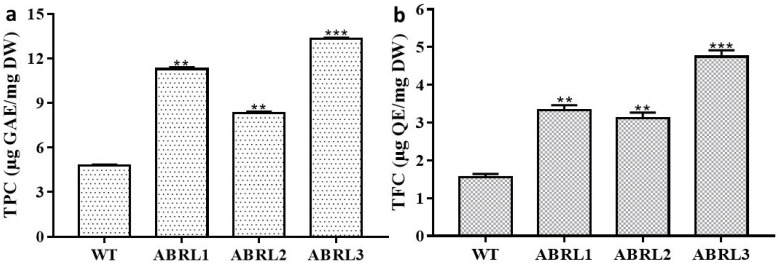
(**a**) total phenolic content (TPC) and (**b**) total flavonoids content (TFC). WT = wild type untransformed *A. bracteosa* plants; ABRL1–3 = transgenic lines 1–3 of *A. bracteosa*. Each value represents mean ± SD (*n* = 3). ** *p* < 0.01 and *** *p* < 0.001 statistical significance.

TPC and TFC were calculated as μg of gallic acid and quercetin equivalents/mg dry extract by using calibration curves of gallic acid (y = 0.0163x − 0.0130, R^2^ = 0.9983) and quercetin (y = 0.0517x + 0.0172, R^2^ = 0.9991), respectively (Figure 1). In this study, ABRL3 has maximum quantity of phenolics (13.39 ± 2 µg GAE/mg DW) followed by ABRL1 (11.28 ± 1 µg GAE/mg DW), and ABRL2 (8.31 ± 1.5 µg GAE/mg DW). Whereas WT contains minimum amount of phenolics (4.803 ± 0.04 µg GAE/mg DW). Similarly, TFC ranges from the highest value in ABRL3 (4.75 ± 0.16 µg QE/mg DW) to the lowest value in WT (1.55 ± 0.08 µg QE/mg DW).

#### 2.3.2. RP-HPLC

**Figure 2:**
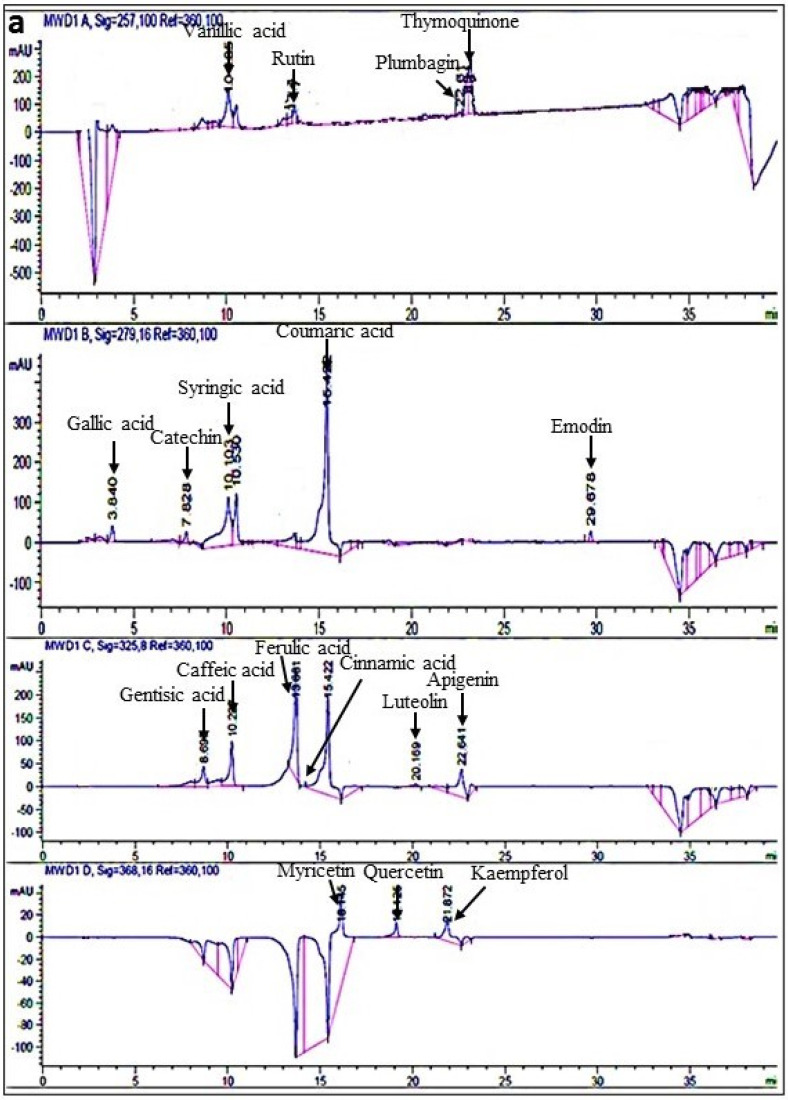

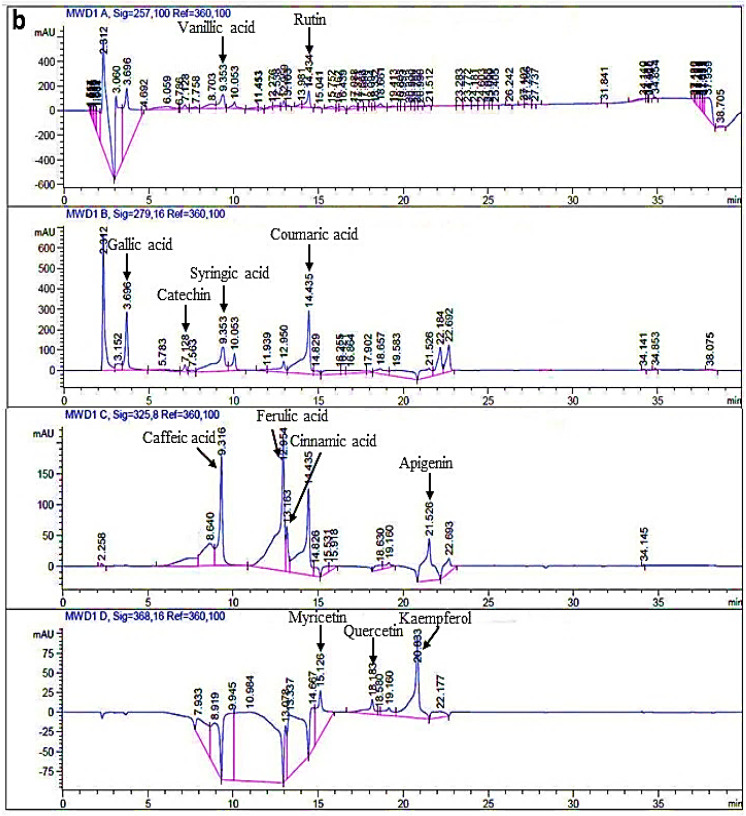
RP−HPLC chromatograms for quantification of polyphenols in crude extracts of *Ajuga bracteosa*. (**a**) mixed standard compounds. (**b**) crude extract of transgenic line 3 of *A. bracteosa* (ABRL3).

Among the eighteen polyphenolic standards tested through RP-HPLC, twelve were detected in all extracts of *A. bracteosa*. Kaempferol was most abundant in all transgenic samples ranging from 78.6 ± 5 µg/mg in ABRL2 to 101.26 ± 6 µg/mg in ABRL3. WT contained the highest amount of ferulic acid (75.55 ± 3 µg/mg) and the lowest amount of rutin (0.63 ± 0.5 µg/mg). Cinnamic acid was least abundant in all transformed lines with 4.36 ± 0.5 µg/mg in ABRL1, 5.47 ± 0.2 µg/mg ABRL2, and 6.09 ± 0.3 µg/mg in ABRL3 (Table 3). Overall, ABRL3 has a predominantly higher content of all polyphenols screened in this study as represented by chromatogram in Figure 2.

#### 2.3.3. Total Antioxidant Capacity (TAC) and Total Reducing Power (TRP)

**Figure 3:**
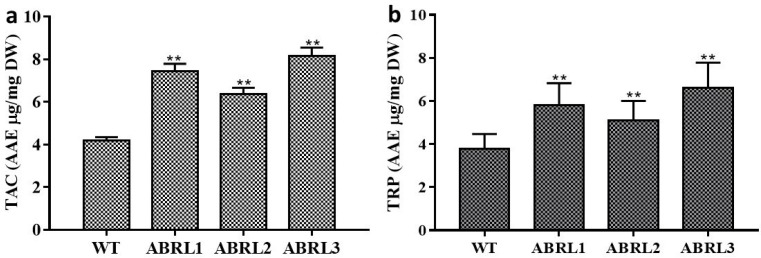
(**a**) total antioxidant capacity (TAC) and (**b**) total reducing power (TRP). WT = wild type untransformed *A. bracteosa* plants; ABRL1–3 = transgenic lines 1–3 of *A. bracteosa*. Each value represents mean ± SD (*n* = 3). ** *p* < 0.01 statistical significance.

Total antioxidant capacity (TAC) and total reducing power (TRP) of samples against ascorbic acid equivalent are given in Figure 3. Results showed that ABRL3 exhibited the maximum total antioxidant capacity (8.16 ± 1 μg AAE/mg DW) while minimum antioxidant capacity was shown by WT (4.18 ± 0.16 μg AAE/mg DW). TAC was found to decrease in the order ABRL3 > ABRL1 > ABRL2 > WT. All the transgenic lines showed significantly increased antioxidant activity (*p* < 0.01) compared to wild plants. Data also shows that ABRL3 exhibited the highest reduction power of 6.60 ± 1.17 μg AAE/mg DW followed by 5.8 ± 1 μg AAE/mg DW in ABRL1. TRP followed the as that of TAC in all plant extracts. However, TRP was significantly higher (*p* < 0.01) in transgenic regenerants than wild control plants.

### 2.4. In Vitro Antioxidant Assays

#### 2.4.1. DPPH Radical Scavenging Assay

**Figure 4:**
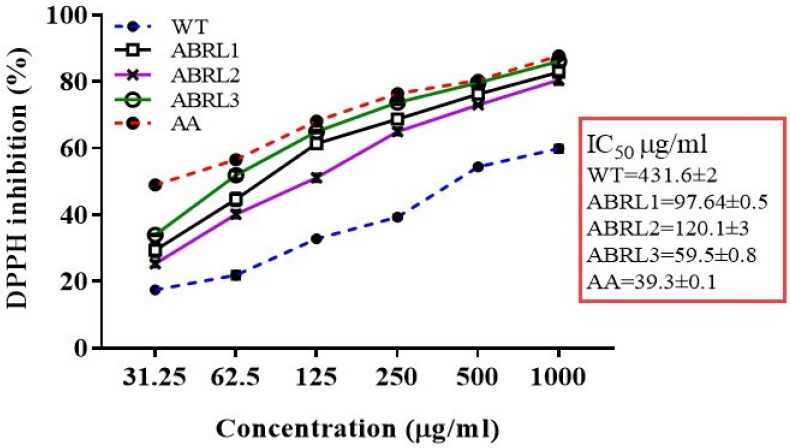
DPPH scavenging assay of crude extracts of *A. bracteosa* along with IC_50_ values. WT = wild type untransformed *A. bracteosa* plants; ABRL1–3 = transgenic lines 1–3 of *A. bracteosa*; AA = ascorbic acid.

The percent scavenging activity of the extracts were evaluated using the DPPH free radical scavenging assay and IC_50_ values were calculated (Figure 4). IC_50_ values varied in a concentration-dependent manner however, all extracts showed higher IC_50_ values than ascorbic acid (IC_50_ 39.3 ± 1 µg/mL), used as the positive control. The minimum IC_50_ values were exhibited by ABRL3 (59.5 ± 0.8 μg/mL) followed by ABRL1 (97.64 ± 0.5 μg/mL). Overall, order of IC_50_ values was ABRL3 < ABRL1 < ABRL2 < WT. The DPPH radical scavenging activity of different extracts presented good correlation with TPC (R^2^ = 0.9627 ***, *p* < 0.001) and moderate correlation with TFC (R^2^ = 0.7627 **, *p* < 0.01) as shown in Table 4.

#### 2.4.2. Hydroxyl Ion Scavenging Assay

**Figure 5:**
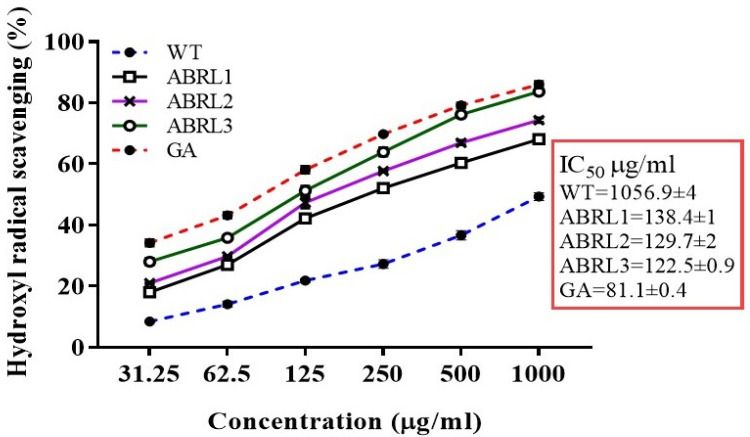
Hydroxyl ion scavenging activity of *A. bracteosa*. WT = wild type untransformed *A. bracteosa* plants; ABRL1–3 = transgenic lines 1–3 of *A. bracteosa*; GA = gallic acid.

All extracts of *A. bracteosa* scavenged ^•^OH radicals while lowest IC_50_ values were recorded for ABRL3 (122.5 ± 0.90 μg/mL) and ABRL2 (129.7 ± 2 μg/mL) followed by ABRL1 (138.4 ± 1 μg/mL), whereas the highest IC_50_ was observed for WT (1056.9 ± 4 μg/mL). IC_50_ of all extract samples were significantly different from the standard gallic acid (81.1 ± 4 μg/mL) as given in Figure 5. A highly significant correlation was observed with TPC (R^2^ = 0.9312 ***, *p* < 0.001) and moderate correlation with TFC (R^2^ = 0.8158 **, *p* < 0.01) (Table 4).

#### 2.4.3. Ferrous Ion Chelating Activity

**Figure 6:**
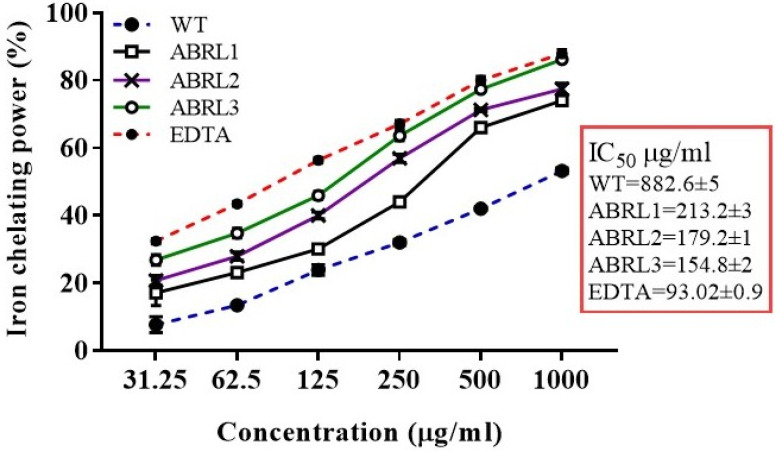
Iron chelating power of *A. bracteosa*. WT = wild type untransformed *A. bracteosa* plants; ABRL1–3 = transgenic lines 1–3 of *A. bracteosa*; EDTA = ethylenediaminetetraacetic acid.

In this study, the finest values for IC_50_ were exhibited by ABRL3 (154.8 ± 2 μg/mL) followed by ABRL2 (179.2 ± 1 μg/mL). Overall, order of IC_50_ of ABRL3 < ABRL2 < ABRL1 < WT was observed (Figure 6). The iron chelating activity of various extracts showed good correlation with TPC (R^2^ = 0.9159 ***, *p* < 0.001) and moderate correlation with TFC (R^2^ = 0.8243 **, *p* < 0.01) as given in Table 4.

### 2.5. In Vivo Assays in BALB/c Mice

#### 2.5.1. Analgesic Activity

**Figure 7:**
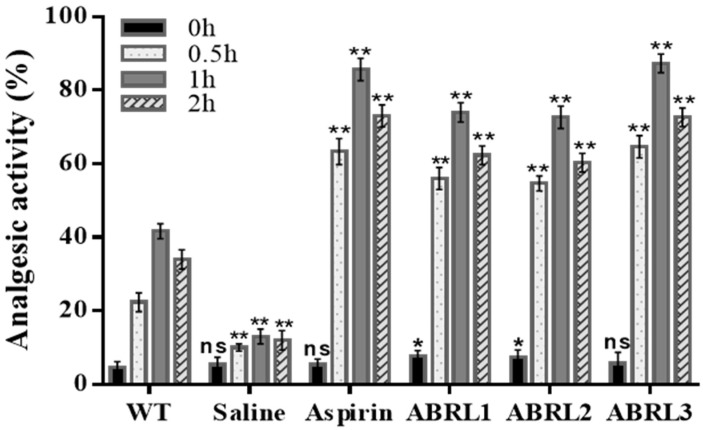
In vivo analgesic activity of *Ajuga bracteosa* crude extracts. WT = wild type untransformed *A. bracteosa* plants; ABRL1–3 = transgenic lines 1–3 of *A. bracteosa*. Each value represents mean ± SD (*n* = 5). ns shows non-significant values, * *p* < 0.05, and ** *p* < 0.01 statistically significant data.

The crude extracts of transgenic *A. bracteosa* plants showed a delayed latency period and increased analgesic activity in BALB/c mice. Aspirin (positive control) and crude extracts displayed a time-dependent activity on a hot plate by suppressing nociceptor activity in mice (Figure 7). Maximum activity was observed after 1 h of oral dose in ABRL3 (87.3 ± 3%) followed by aspirin (85.66 ± 4%). Whereas normal saline-treated mice (negative control) produced the least significant analgesic effect (13 ± 4%). ABRL1 and ABRL2 also demonstrated increased activity (76 ± 3% and 74 ± 2%) compared to the wild-type control group (41 ± 3%).

#### 2.5.2. Anti-Inflammatory Activity

**Figure 8:**
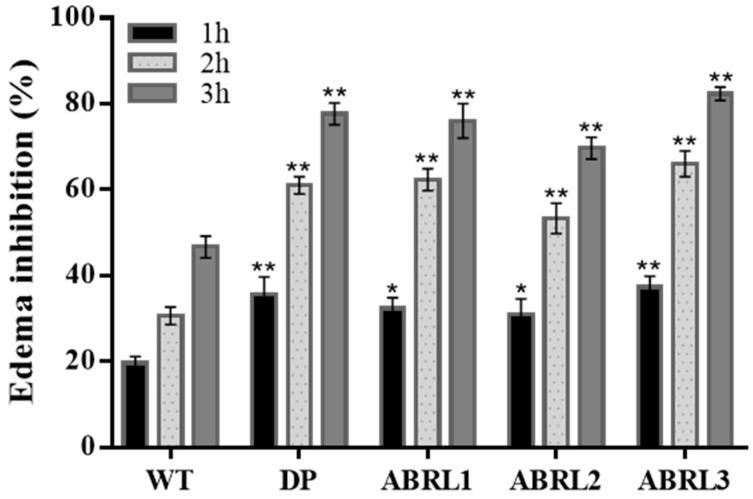
In vivo anti-inflammatory activity of *Ajuga bracteosa* crude extracts. DP = diclofenac potassium; WT = wild type untransformed *A. bracteosa* plants; ABRL1–3 = transgenic lines 1–3 of *A. bracteosa*. Each value represents mean ± SD (*n* = 5). * *p* < 0.05, and ** *p* < 0.01 statistically significant data.

Pain and inflammation are often linked to each other. Therefore, crude extracts were also tested for anti-inflammatory activity against carrageenan-induced hind paw edema in BALB/c mice. Observations were made after 1 h, 2 h, and 3 h of oral dose for edema treatment. Diclofenac potassium was used as a positive drug and showed 77.6 ± 3% activity after 3 h post dosage. ABRL3 revealed the highest anti-inflammatory activity (82.3 ± 2 %) while WT manifested the lowest activity (46.6 ± 2%) among all tested samples (Figure 8).

#### 2.5.3. Antidepressant Activity

**Figure 9:**
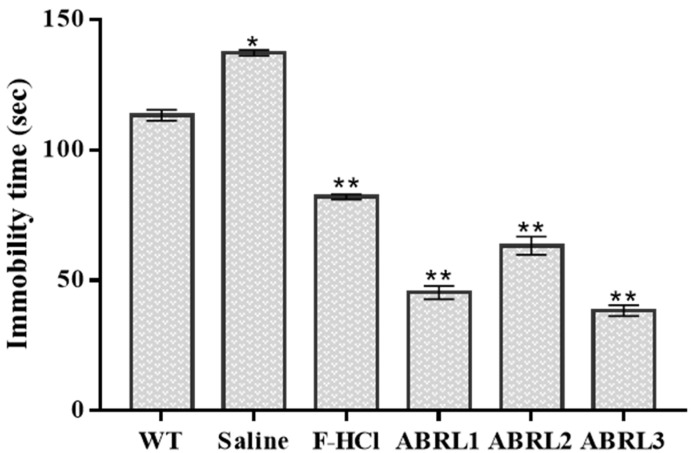
In vivo anti-depressant activity of *Ajuga bracteosa* crude extracts. F-HCl = fluoxetine HCl; WT = wild type untransformed *A. bracteosa* plants; ABRL1–3 = transgenic lines 1–3 of *A. bracteosa*. Each value represents mean ± SD (*n* = 5). * *p* < 0.05, and ** *p* < 0.01 statistically significant data.

The potential antidepressant activity of *A. bracteosa* crude extracts was evaluated in mice by tail suspension test and results are displayed in Figure 9. All transgenic lines exhibited enhanced antidepressant activity compared to wild-type and positive control (Fluoxetine-HCl). ABRL3 demonstrated the lowest immobility time of 38.3 ± 2 s followed by 45.7 ± 3 s in ABRL1 and 63.3 ± 4 s in ABRL2.

#### 2.5.4. Anticoagulant Activity

**Figure 10:**
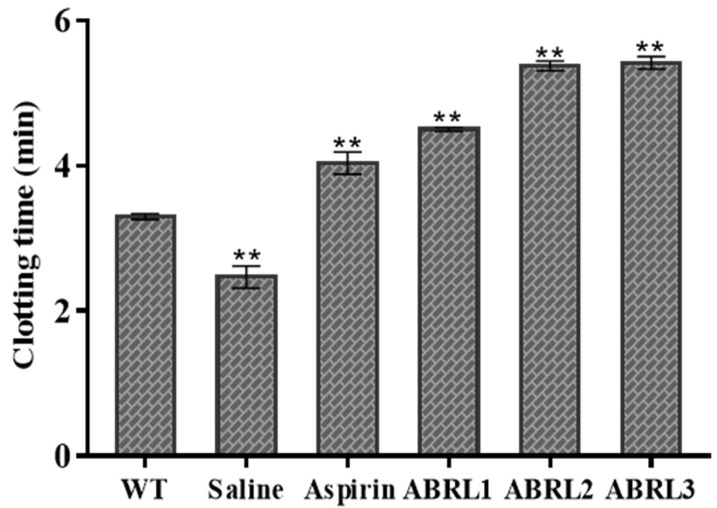
In vivo anti-coagulant activity of *Ajuga bracteosa* crude extracts. WT = wild type untransformed *A. bracteosa* plants; ABRL1–3 = transgenic lines 1–3 of *A. bracteosa*. Each value represents mean ± SD (*n* = 5). ** *p* < 0.01 statistically significant data.

Anticoagulant activity of wild type and transgenic lines of *A. bracteosa* is shown in Figure 10. Transgenic lines delayed blood clotting from 2.46 min in negative control to 4.51 min, 5.40 min, and 5.41 in ABRL1, ABRL2, and ABRL3 treated mice, respectively. WT crude extracts were least effective among all plant extracts with a clotting time of 3.3 min.

## 3. Discussion

The plant kingdom consists of countless species with several uses in medicines. They contain valuable secondary metabolites demonstrating anti-inflammatory, anticancer, antioxidant antidiabetic, and/or antimicrobial properties [16]. The growing demand for these plant secondary metabolites forces the use of new green biotechnology tools to create new, more productive in vitro transgenic plant cultures [17]. This study reports the examination of the potential role of *rolABC* genes in increasing the production of secondary metabolites and compares the pharmaceutical efficacy of transgenic regenerants and in vitro grown untransformed *A. bracteosa* plants.

Minerals are requisite for disease resistance and normal physiology of the human body; however, high concentrations could be a health risk. Major elements including sodium, potassium, calcium, and magnesium are involved in protein synthesis, nerve transmission, bone development, muscle contraction, and enzyme activation [18]. Here we found that all studied elements were below the maximum permissible limit as recommended by WHO and European pharmacopeia [19]. Among 10 detected elements, potassium was most abundant in all samples. However, the Na/K ratio was below 1 which is required to maintain normal blood pressure [20]. Only minute amounts of 6 trace elements were detected. These trace elements have well reported anti-inflammatory, antianemia, antidiabetic, and anticancer properties [21].

Next, phytochemical analysis was carried out to investigate the effects of transformation on secondary metabolite production. Qualitative assays screened several medicinally active molecules in *A. bracteosa* based on their polarities. Considerable amounts of alkaloids, phenolics, flavonoids, and glycosides were present in all extracts. These phytoconstituents possess antimicrobial, antioxidant, anti-inflammatory, and anticancer properties [22]. Saponins and tannins used as astringents, hepaprotective, cardioprotective, and anticancer agents [23] were present in moderate quantities. The presence of terpenoids in transgenic plants can impart antioxidant, antibacterial, antiviral, chemoprotective, and neuroprotective characters [24,25] to these transgenics.

The antioxidant activity of phenolics and flavonoids primarily depends on the presence of hydroxyl groups [26]. In a previous study on *A. bracteosa*, the highest amount of phenolic content was found in crude extracts prepared in more polar solvents (10.75 ± 0.70 μg GAE/mg DW of methanolic extracts) than the non-polar solvent systems (0.33 ± 0.0 μg GAE/mg DW demonstrated by *n*-hexane extract). A similar pattern of results was obtained for flavonoid content [10]. The current study demonstrated significantly higher values of TPC and TFC in transformed regenerants as compared to untransformed plants. Our observations are in accordance with the studies reporting enhanced TPC and TFC of *Artemisia dubia*, *A. annua*, and *Lactuca sativa* plants transformed with *rolABC* genes [27,28,29].

RP-HPLC delineated a marked surge in polyphenols of transformed plants than their untransformed counterparts. These differences support our previous findings indicating that *rolABC* genes enhanced the expression of biosynthetic pathway genes in transformed regenerants of *A. bracteosa* [15]. We found that ABRL3 presented the highest expression of β-hydroxy β-methylglutaryl-CoA reductase (HMGR), farnesyl diphosphate synthase (FDS), 4-hydroxy-3-methyl-but-2-enyl pyrophosphate synthase (HDS), and phenylalanine ammonia-lyase (PAL) genes owing to the significant phenolic content and antioxidant activity of this line. Higher expression of the metabolic pathway genes can easily be correlated to the higher production of phytoecdysteroids and hence the polyphenols contents. These polyphenols are reported to prevent aging and age-related disorders such as cancer, cardiac malfunctions, diabetes, neurodegenerative problems, and inflammatory disorders [3]. Their protective mechanisms involve suppression of oxidative stress, maintenance of nuclear factor kappa B (NF-κB) and interleukins (IL-1β), activation of gluconeogenesis, inhibition of angiogenesis, and cyclooxygenases (COX-1 and COX-2) [30]. Our results show that six polyphenol markers, namely, plumbagin, thymoquinone, catechin, emodin, gentisic acid, and luteolin were below the detection limit in all crude extracts. However, some earlier studies [10] reported catechin in ethyl acetate and aqueous fractions of *A. bracteosa*. These findings are likely to be related to the diverse nature of metabolites and polarity in different solvent systems [31].

All extracts were evaluated for TAC, TRP, and antioxidant activity through DPPH, hydroxyl radical scavenging, and iron-chelating power assays. Transgenic lines fared better for reducing power and antioxidant activity than untransformed plants. A similar pattern of results was found in *Artemisia annua* [32] and lettuce [33] plants transformed with different *rol* genes. Previously, Kayani et al. (2016) [8] also determined the phytochemical content and antioxidant activity of naturally growing *A. bracteosa* plant extracts in different solvent systems. They observed that the aerial and root extracts of *A. bracteosa* prepared in methanol and chloroform represented the best TPC, TFC, TAC, TRP, and free radical scavenging among all solvents. Moreover, among 15 different extracts of *A. bracteosa*, the maximum total reducing power (23.90 ± 0.70 μg AAE/mg DW) and total antioxidant capacity (11.30 ± 0.80 μg AAE/mg DW) were exhibited by methanol extract with superlative percent extract recovery (17.50 ± 0.80% *w*/*w*) [10]. Similarly, Hafeez et al. (2017) [34] reported potential DPPH radical scavenging activity of *A. bracteosa* root extracts along with antibacterial and antidiabetic activity in both polar and non-polar solvents.

To find the main components responsible for the antioxidant activity of the crude extracts we developed a correlation with total phenolic content and total flavonoid content. We observed a significant positive correlation between antioxidant activities and plant phenolics. Whereas moderate correlation with TFC suggests that reported antioxidant properties are attributed to higher phenolics than flavonoids content [10].

Despite the low bioavailability of dietary polyphenolics and phytoecdysteroids, most of their biometabolites display higher biological activity both in vitro and in vivo. These metabolites account for the health benefits of their parent phytochemicals [5,35]. Considering this, we tried to demonstrate the in vivo pharmacological potential of *A. bracteosa*.

Hot plate assay is a simple and sensitive method to assess anti-nociceptive drugs to relieve pain [36]. The results of analgesic activity showed that transgenic plant extracts protected mice from visceral pain and produced comparable effects to standard drug aspirin. Nonsteroidal anti-inflammatory drugs (NSAIDs) can be used to treat inflammation associated with pain. However, their overuse can lead to serious side effects. Therefore, herbal products are used as an alternative to antagonize inflammatory agents [36]. Blood brain barrier (BBB) is the key target for the therapeutic delivery of central nervous system (CNS) drugs. Inflammation and neurodegeneration are often associated in neurological disorder [37]. Different studies suggest that polyphenols, alkaloids, terpenoids, steroids, and lignans can attenuate the inflamed and damaged BBB thus acting as curative agent against neurovegetative disorders [38,39] Previously, ferulic acid was physiologically detected in the cerebrospinal fluid (CSF) of mice and rat [40,41]. Interestingly, a recent study demonstrated the presence of non-endogenous caffeic acid in the human CSF. These studies confirm that plant phenolics can cross the BBB in humans and reach the neurons and microglia [42]. However, the exact mechanisms by which they may permeate the BBB are not fully understood. Additionally, little is known about their further metabolism in the brain [43]. Different studies suggest that with anolides and phenolics present in *A. bracteosa* inhibit the activity of prostaglandins and COX [44,45]. Terpenoids, phytoecdysteroids, and phenolics attribute to anti-depressant and anticoagulant properties of transformed *A. bracteosa* plant extracts. Thus, this plant can be a potent agent to treat neurodegenerative and thrombolytic disorders [8,27]. Our findings are in agreement with former results [33] verifying that *rol* genes increased the pharmaceutical value of methanolic extracts of transformed lettuce in rats.

## 4. Materials and Methods

### 4.1. Source of Plant

The plant material was collected from the premises of Quaid-i-Azam University, Islamabad, Pakistan. These plants were identified by Prof. Dr. Rizwana Aleem Qureshi (Taxonomist), Department of Plant Sciences, Quaid-i-Azam University, Islamabad. A voucher specimen number (HPM-460) was deposited in the herbarium of Quaid-i-Azam University. Fresh green plants were surface sterilized, and tissue cultured on Murashige and Skoog (MS) medium. Transgenic hairy roots were generated through *rolABC* containing *A. rhizogenes* mediated transformation. Intact plants were regenerated from hairy roots by following the previously optimized method in our lab [46]. Polymerase chain reaction (PCR) was performed for the confirmation of transformation and stable integration of *rol* genes in transformed roots and regenerants [15,46]. Untransformed tissue cultured plants and transgenic regenerants of *A. bracteosa* plants were used in this study.

### 4.2. Elemental Analysis

For the digestion of leaf samples, an earlier reported method [47] was employed with heated at 550 °C for 4 h and then cooled at room temperature. Then Nitric acid (6 M; 10 mL) was added for acid digestion. After filtration, the solution was diluted up to a 25 mL mark with deionized water. Reference standards of 12 micro and macro elements (Sigma-Aldrich, Burlington, MA, USA) were used to quantify the essential elements and to find the possible accumulation of hazardous heavy metals in *A. bracteosa*. Estimation of all elements was carried out on a Fast Sequential Atomic Absorption Spectrometer (Varian 240AA FS, Melbourne, Australia). The operating parameters for working elements were optimized according to the manufacturer’s recommendations.slight modifications. Powdered leaf samples were weighed (100 mg each) and heated in an oven at 110 °C in a china dish for the removal of moisture. The dried samples were.

### 4.3. Crude Extracts Preparation for Biological Activities

Aerial parts of three independent transgenic lines (ABRL1, 2, and 3) and in vitro grown untransformed *A. bracteosa* plants (WT) were shade dried after rinsing with water. Leaves were ground and 1 g powdered material was allowed to set in a mixture of methanol: chloroform (1:1; 5 mL). After 1 h the mixture was sonicated for 10 min followed by 20 min of shaking and the process was repeated three times. Finally, the plant extracts were filtered, and pooled filtrates were dried, weighed, and stored at room temperature for further analysis.

### 4.4. Phytochemical Profiling

#### 4.4.1. Qualitative Assays

Crude extracts of *A. bracteosa* were evaluated for the presence of major families of secondary metabolites such as alkaloids, glycosides, flavonoids, phenols, tannins, saponins, terpenoids, coumarins, betacyanin (β-cyanin), anthocyanin, and sterols using standard quality procedures based on coloring reaction and/or precipitation [48].

#### 4.4.2. Quantitative Assays

##### Estimation of Total Phenolics and Flavonoids Content

The total phenolic content (TPC) was calculated by Folin–Ciocalteu (FC) assay using gallic acid as standard [49]. Briefly, 20 mg of each dried extract was dissolved in 1 mL of DMSO. Gallic acid (1 mg, Merck, Kenilworth, NJ, USA) was dissolved in 1 mL of DMSO and further diluted. In this experiment, 5 µL of plant extract was mixed with 98 µL of 10 times diluted FC reagent in 96 well plate (Thermo Scientific, Waltham, MA, USA). After 5 min, 98 µL of 6% sodium carbonate was added and incubated at 25 °C for 90 min. Finally, the absorbance was measured at 630 nm with a microtiter plate reader (Elx 800, BioTek, Winooski, VT, USA). TPC was expressed as gallic acid equivalents (µg GAE/mg dry weight of plant extract). Total flavonoid content (TFC) was determined by the already optimized colorimetric method [27] with slight modifications. 5 µL of each extract (20 mg/mL) was mixed with equal volumes of potassium acetate (1 M) and 10% aluminum chloride (20 µL each). Then, 155 µL of distilled water was subsequently added and the mixture was left at 37 °C for half an hour. After incubation, absorbance was determined at 405 nm and TFC was expressed as quercetin equivalents (µg QE/mg extract).

##### Quantification of Polyphenols by RP-HPLC

For RP-HPLC analysis, polyphenols were extracted from aerial parts of wild plant and transgenic lines according to the previously reported procedure [50]. HPLC-DAD system (Agilent Technology, Ratingen, Germany) was attached to an analytical column (Sorbex RXC-8) with the dimensions, 5 μm, 4.6 by 250 mm, for separation of polyphenols. This separation was achieved using two mobile phases; water:methanol:acetonitrile:acetic acid (85:10:5:1) were present in mobile phase A while mobile phase B included methanol:acetonitrile:acetic acid (60:40:1). The process was operated by regulating the gradient program as follows; (t (min), % B); (0, 50), (20, 50), (25, 100), and (40, 100) with 1 mL/min flow rate at 350 bars. Then, 20 μL of each crude extract and standard was injected. Identification and quantification of polyphenols were carried out by comparison of the peaks of each metabolite with particular retention time in the standards.

### 4.5. In Vitro Assays

#### 4.5.1. Total Antioxidant Capacity Assay

Total antioxidant capacity (TAC) assay of plant extracts was evaluated by the method of Phatak and Hendre [51] with some modifications. An aliquot of 10 μL of samples was mixed with 190 μL of molybdenum (IV) solution (4 mM ammonium molybdate, 0.6 M sulphuric acid, and 28 mm sodium phosphate) and incubated at 95 °C. After 90 min, absorbance was measured at 630 nm and TAC was reported as ascorbic acid equivalent (μg AAE/mg DW).

#### 4.5.2. Total Reducing Power (TRP)

Reducing power activity was estimated according to the method of Wintola and Afolayan [52] with some modifications. In this assay, 125 µL of all extracts and standards were thoroughly mixed with phosphate buffer (0.2 M; pH 6.6) and 1% potassium ferricyanide (313 µL each). After 20 min of incubation at 50 °C, 313 µL of trichloroacetic acid (10%; TCA) was added. This mixture was centrifuged at 3000 rpm for 10 min. the upper layer (100 µL) was mixed with an equal volume of distilled water and 20µL of ferric chloride (1%). Absorbance was measured at 630 nm and results were recorded as ascorbic acid equivalent.

#### 4.5.3. Antioxidant Assays

##### Diphenyl-2-Picryl-Hydrazyl (DPPH) Radical Scavenging Activity

Antiradical activities of potent antioxidants from crude extracts were detected by the previously reported DPPH method [33]. Ascorbic acid (1 mg, Merck, Darmstadt, Germany) dissolved in 1 mL of DMSO was used as a positive control. The assay was performed by mixing 150 µL of freshly prepared DPPH solution (0.1 mM) with 50 µL of different dilutions of extracts. The resultant mixture was incubated in dark at 37 °C for 1 h. Decolorization of DPPH indicated the antioxidant activity of test samples. Consequently, a reduction in absorbance was recorded at 515 nm. The radical scavenging activity of DPPH was calculated by the following Formula (1):

(1) $$\mathrm{Percent}\;\mathrm{radical}\;\mathrm{scavenging}=\left[\frac{\mathrm{Absorbance}\;\mathrm{of}\;\mathrm{control}-\mathrm{Absorbance}\;\mathrm{of}\;\mathrm{sample}}{\mathrm{Absorbance}\;\mathrm{of}\;\mathrm{control}}\right]\times 100$$

##### Hydroxyl Ion (OH) Scavenging Assay

The OH scavenging potential of extracts was determined by classical thiobarbituric acid (TBA) and deoxyribose-based method [53]. For this assay, a 2.8 mM solution of 2-deoxyribose (*w*/*v*) was prepared in phosphate buffer (50 Mm; pH 7.4). Then, 125 µL of 2-deoxyribose (2.8 mM), 25 µL EDTA (0.1 M), 25 µL ferric chloride (100 M), 25 µL of 0.2 M H_2_O_2_, and 25 µL of ascorbic acid (0.3 M) were mixed with 100 µL of plant extract. The whole mixture was kept at 37 °C for 1 h. Afterward, 250 µL of 2.8% TCA and 1.25 mL of 1% TBA prepared in sodium hydroxide (50 mM) were added. The resulting solution was heated for 15 min in a water bath and then placed for cooling. Then absorbance was measured at 540 nm and OH radical scavenging activity was calculated through the following Formula (2):

(2) $$\mathrm{Percent}\;\mathrm{OH}\;\mathrm{radical}\;\mathrm{scavenging}=\left[\frac{\mathrm{Absorbance}\;\mathrm{of}\;\mathrm{control}-\mathrm{Absorbance}\;\mathrm{of}\;\mathrm{sample}}{\mathrm{Absorbance}\;\mathrm{of}\;\mathrm{control}}\right]\times 100$$

In this study, gallic acid was used as positive control while DMSO was a negative control.

##### Chelating Power Assay

The Ferrous ion (Fe^2+^) chelating ability of plant extracts was investigated by the reported method of Dastmalchi et al. [54]. In this method, 100 µL of plant samples were added to 50 µL ferrous chloride (2 mM) and incubated for 5 min. Then 200 µL ferrozine (5 mM) was added to initiate ferrous-ferrozine complex formation reaction. The mixture was re-incubated for 10 min then absorbance was measured at 540 nm. The ability of plant extracts to inhibit complex formation was measured by the following Formula (3):

(3) $$\mathrm{Percent}\;\mathrm{iron}\;\mathrm{chelating}\;\mathrm{power}=\left[\frac{\mathrm{Absorbance}\;\mathrm{of}\;\mathrm{control}-\mathrm{Absorbance}\;\mathrm{of}\;\mathrm{sample}}{\mathrm{Absorbance}\;\mathrm{of}\;\mathrm{control}}\right]\times 100$$

### 4.6. In Vivo Biological Activities

#### 4.6.1. Test Animals

For the experiment, healthy male BALB/c albino mice (4–6 weeks; 25–30 g) were procured from the NIH, Islamabad, Pakistan. Test mice were maintained under controlled environmental conditions (23.0 ± 2.0 °C temperature; 60–70% of relative humidity; 12 h light/dark cycle) in the Primate facility Quaid-i-Azam University (QAU), Islamabad, Pakistan. All the mice were fed with a pellet diet and had free access to water ad libitum. All the experiments were approved by the Animal Ethical Committee, QAU (BEC-FBS-QAU2019-157).

#### 4.6.2. Experimental Design

Plant extracts and standard compounds were prepared in normal saline (0.9% NaCl) and administered orally according to mice body weight. In these experiments, 30 male mice were randomly divided into 6 groups as follows:

Normal control group: 200 mg/kg normal saline.

Positive control group: 10 mg/kg standard drugs.

Group-1: 200 mg/kg crude extract of a wild-type plant (WT).

Group-2: 200 mg/kg crude extract of ABRL1.

Group-3: 200 mg/kg crude extract of ABRL2.

Group-4: 200 mg/kg crude extract of ABRL3.

#### 4.6.3. Acute Toxicity Test

Before performing in vivo experiments, animals were tested for toxic effects of *A. bracteosa* plant extracts. The oral administration of plant crude extracts (up to 1 g/kg) proved to be non-toxic and did not produce mortality for the next 24 h. Guidelines 425 of the Organization for Economic Corporation and Development (OECD) were strictly followed during the study.

#### 4.6.4. Analgesic Activity (Hot-Plate Method)

Analgesic activity was determined by a previously optimized method [55] with slight modifications. Before any dosage, mice were placed on a hot plate (set at a temperature of 55 °C) to determine initial reaction time based on stimulation of pain by heat for a cut-off time of 30 s. After 1 h of dosage, a licking or jumping reaction was observed on a hot plate at different time intervals of 1, 2, and 3 h. The standard drug, aspirin was used as positive control while normal saline was a negative control.

#### 4.6.5. Anti-Inflammatory Activity

The anti-inflammatory activity of *A. bracteosa* crude extracts was assessed through the carrageenan-induced hind paw edema model [56]. Diclofenac potassium was used as a positive control. Then, 1 mL/kg of freshly prepared λ-carrageenan (1% *w*/*v* in normal saline) was injected into mice paw. After 30 min of edema induction, an oral dose of crude extracts and controls was administered. Control reading of paw volume was taken immediately before and after injecting carrageenan into the sub plantar region by using Plethysmometer (UGO Basile 7140, Varese, Italy). Change in paw volume was measured for up to 3 h. Anti-inflammatory activity was calculated as percent edema inhibition through the following Formula (4):

(4) $$\mathrm{Percent}\;\mathrm{edema}\;\mathrm{inhibition}=\left[\frac{\mathrm{Edema}\;\mathrm{volume}\;\mathrm{in}\;\mathrm{control}-\mathrm{Edema}\;\mathrm{volume}\;\mathrm{in}\;\mathrm{test}}{\mathrm{Edema}\;\mathrm{volume}\;\mathrm{in}\;\mathrm{control}}\right]\times 100$$

#### 4.6.6. Anti-Depressant Activity by Tail Suspension Test (TST)

Tail suspension test is a simple and inexpensive method designed by Steru et al. [57] to assess anti-depressant activity in mice. Briefly, after pretreatment with positive drug and crude extracts, each mouse was suspended by the tail for 6 min, using an adhesive tape. The immobility score (in seconds) was recorded during the final 4 min of the experiment. Data collected in TST were expressed as arithmetic means of immobility time for each experimental group.

#### 4.6.7. Anticoagulant Assay

A capillary tube was used to analyze the anticoagulation activity of the crude extracts of *A. bracteosa* [36]. Briefly, after 1 h of oral dosage, the mice’s tail was pricked with a sterile needle, and blood was collected in a capillary tube. These tubes were placed at 37 °C for 10 s. Then a small part of the tube was broken at intervals till fibrin threads appear between two parts of the tube. The time interval between the appearance of blood on mice’s tails and thread formation was the blood clotting time.

### 4.7. Statistical Analysis

All the experiments were performed in triplicates. One-way analysis of variance (ANOVA) was used to find variability between data by Statistix 8.1 (Analytical Software, Tallahassee, FI, USA). IC_50_ values were calculated through GraphPad Prism Software Version 7.0 (GraphPad Prism^®^ Software, Inc., San Diego, CA, USA) by plotting transformed inhibitors vs. log values of inhibition. The correlation between IC_50_ values of antioxidant assays and TPC and TFC was found by GraphPad 7. Here, *p* > 0.05 was considered significant, and data were represented as the mean of 3 values ± SD (Standard Deviation).

## 5. Conclusions

In conclusion, present findings suggest that transgenic *A. bracteosa* plants are a rich source of essential elements and polyphenols. Overall, all transgenic lines showed significant antioxidant, analgesic, anti-inflammatory, antidepressant, and anticoagulant activities as compared to the untransformed wild-type plant extracts. We could see that *rol* genes influenced positively the production of active secondary metabolites and this could be linked to the enhanced activities exhibited by the transgenic plant extracts. This study provides more rationalized scientific reasons for the folklore use of this plant. However, further investigations are needed to understand the underlying protective mechanisms.

## Figures and Tables

**Table 1:** Quantity of different elements in aerial parts of *Ajuga bracteosa*.

Elements (µg/mg)	λ_max_ (nm)	Slit Width (nm)	Samples			
			WT	ABRL1	ABRL2	ABRL3
Sodium	589.0	0.5	2.86 ± 0.2	3.54 ± 0.8 ^b^	3.07 ± 1 ^b^	3.94 ± 2 ^ab^
Potassium	766.5	1.0	8.06 ± 0.3	12.00 ± 1 ^bc^	12.62 ± 3 ^b^	13.09 ± 2 ^a^
Calcium	422.7	0.5	0.50 ± 0.06	1.69 ± 0.5 ^a^	1.32 ± 0.4 ^a^	1.95 ± 0.5 ^a^
Magnesium	285.2	0.5	0.91 ± 0.1	2.15 ± 0.3 ^a^	1.12 ± 0.2 ^b^	2.12 ± 0.7 ^a^
Zinc	213.9	1.0	0.25 ± 0.05	0.40 ± 0.1 ^b^	0.24 ± 0.04 ^c^	0.47 ± 0.03 ^a^
Iron	248.3	0.2	0.25 ± 0.1	0.33 ± 0.2 ^c^	0.46 ± 0.1 ^a^	0.44 ± 0.2 ^a^
Manganese	279.5	0.2	0.004 ± 0.01	0.02 ± 0.01 ^a^	0.01 ± 0.03 ^b^	0.02 ± 0.01 ^a^
Nickel	232.0	0.2	0.09 ± 0.02	0.18 ± 0.1 ^b^	0.19 ± 0.1 ^b^	0.23 ± 0.2 ^a^
Copper	324.8	0.5	0.005 ± 0.01	0.01 ± 0.02 ^b^	0.01 ± 0.03 ^b^	0.02 ± 0.01 ^a^
Chromium	357.9	0.2	0.16 ± 0.03	0.45 ± 0.1 ^a^	0.43 ± 0.2 ^a^	0.36 ± 0.1 ^b^

WT = in vitro grown untransformed *Ajuga bracteosa* plant extract; ABRL1–3 = crude extracts of transgenic lines 1, 2, and 3 of *A. bracteosa*. Data are represented as mean ± SD (*n* = 3). The values with different superscript (a–c) letters show significantly (*p* < 0.05) different means.

**Table 2:** Phytochemical constituents of *Ajuga bracteosa*.

Phytochemicals	Samples			
	WT	ABRL1	ABRL2	ABRL3
Alkaloids	++	++	+++	+++
Glycosides	+	+++	++	+++
Flavonoids	+	++	++	+++
Phenols	++	++	++	++
Tannins	+	+	+	+
Saponins	+	+	++	++
Terpenoids	−	+	+	+
Coumarins	−	−	−	−
β-cyanins	−	−	−	−
Anthocyanin	−	−	−	−
Sterols	−	−	−	−

(+) present, (++) moderate concentration, (+++) high concentration, and (−) absent. WT = in vitro grown untransformed *Ajuga bracteosa* plant extract; ABRL1–3 =crude extracts of transgenic lines 1, 2, and 3 of *A. bracteosa*.

**Table 3:** Polyphenolic composition of crude extracts of *Ajuga bracteosa*.

No.	Compound Name	λ_max_ (nm)	Extracts (µg/mg Dry Extract)			
			WT	ABRL1	ABRL2	ABRL3
1	Vanillic Acid	257	8.98 ± 1	15.87 ± 3 ^a^	15.49 ± 2 ^a^	16.33 ± 1 ^a^
2	Rutin	257	0.63 ± 0.5	4.49 ± 1 ^c^	9.24 ± 2 ^b^	14.86 ± 2 ^a^
3	Plumbagin	257	Nd	Nd	Nd	Nd
4	Thymoquinone	257	Nd	Nd	Nd	Nd
5	Gallic Acid	279	4.59 ± 0.3	14.99 ± 2 ^ab^	15.01 ± 3 ^a^	16.67 ± 1 ^a^
6	Catechin	279	Nd	Nd	Nd	Nd
7	Syringic Acid	279	10.79 ± 0.8	13.93 ± 2 ^b^	12.41 ± 1 ^b^	17.78 ± 3 ^a^
8	Coumaric Acid	279	1.92 ± 0.7	15.39 ± 3 ^a^	14.02 ± 1 ^ab^	23.45 ± 2 ^a^
9	Emodin	279	Nd	Nd	Nd	Nd
10	Gentisic Acid	325	Nd	Nd	Nd	Nd
11	Caffeic Acid	325	13.39 ± 2	25.51 ± 3 ^b^	22.01 ± 2 ^b^	30.18 ± 4 ^a^
12	Ferulic Acid	325	75.55 ± 3	77.17 ± 4	76.86 ± 4	78.05 ± 3
13	Cinnamic Acid	325	3.19 ± 0.7	4.36 ± 0.5 ^c^	5.47 ± 0.2 ^b^	6.09 ± 0.3 ^a^
14	Luteolin	325	Nd	Nd	Nd	Nd
15	Apigenin	325	8.20 ± 2	20.84 ± 5 ^bc^	23.12 ± 3 ^b^	32.29 ± 4 ^a^
16	Myricetin	368	4.14 ± 0.7	13.66 ± 3 ^a^	11.6 ± 2 ^b^	13.37 ± 4 ^a^
17	Quercetin	368	4.68 ± 0.3	6.44 ± 0.8 ^c^	7.52 ± 1 ^bc^	9.19 ± 0.5 ^a^
18	Kaempferol	368	17.6 ± 2	83.9 ± 4 ^b^	78.6 ± 5 ^b^	101.26 ± 6 ^a^

WT = in vitro grown untransformed *Ajuga bracteosa* plant extract; ABRL1–3 = crude extracts of transgenic lines 1, 2, and 3 of *A. bracteosa*. Data being represented as mean ± SD (*n* = 3). The values with different superscript (a–c) letters show significantly (*p* < 0.05) different means.

**Table 4:** Correlation of IC_50_ values of different antioxidant activities of *A. bracteosa* with total phenolic and total flavonoid contents.

Antioxidant Activities	Correlation R^2^	
	**TPC**	**TFC**
DPPH Radical Scavenging Activity	0.9627 ***	0.7627 **
Hydroxyl Ion Scavenging Assay	0.9312 ***	0.8158 **
Iron Chelating Power	0.9159 ***	0.8243 **

Columns with different superscripts are significantly different: ** shows *p* < 0.01 while *** shows *p* < 0.001. TPC = total phenolic content; TFC = total flavonoid content.

## Data Availability

The datasets used and/or analyzed during the current study are available from the corresponding author on reasonable request.

## Sample Availability

Samples of plant material are available from the authors.
