# Correction: Rubnawaz et al. Polyphenol Rich *Ajuga bracteosa* Transgenic Regenerants Display Better Pharmacological Potential. *Molecules* 2021, *26*, 4874

**DOI:** 10.3390/molecules27092952

**Published:** 2022-05-05

**Authors:** Samina Rubnawaz, Waqas Khan Kayani, Nosheen Akhtar, Rashid Mahmood, Asif Khan, Mohammad K. Okla, Saud A. Alamri, Ibrahim A. Alaraidh, Yasmeen A. Alwasel, Bushra Mirza

**Affiliations:** 1 Department of Biochemistry, Faculty of Biological Sciences, Quaid-i-Azam University, Islamabad 45320, Pakistan; bushramirza@qau.edu.pk; 2 Department of Biotechnology, Faculty of Sciences, University of Kotli, Azad Jammu and Kashmir 11100, Pakistan; wkkayani@gmail.com; 3 Department of Biological Sciences, National University of Medical Sciences, Rawalpindi 46000, Pakistan; nosheenakhtar@numspak.edu.pk; 4 Drugs Control & Traditional Medicines Division, National Institute of Health, Islamabad 45320, Pakistan; alrashidoon@gmail.com; 5 Institute of Biological Sciences, Faculty of Sciences, University of Malaya, Kuala Lumper 50603, Malaysia; asif.khan.qau@gmail.com; 6 Botany and Microbiology Department, College of Science, King Saud University, Riyadh 11451, Saudi Arabia; malokla@ksu.edu.sa (M.K.O.); saualamri@ksu.edu.sa (S.A.A.); ialaraidh@ksu.edu.sa (I.A.A.); Yasmeen@ksu.edu.sa (Y.A.A.)

The authors of this paper [1] have agreed that they would like to add Waqas Khan Kayani as a co-author, as he contributed to the generation of whole plants from transgenic hairy roots of *Ajuga bracteosa* and to the writing the original manuscript. The authors apologize for any inconvenience caused and state that the scientific conclusions are unaffected. The original publication has also been updated.
